# The Importance of Fetoplacental Doppler Velocimetry for Fetal Surveillance During General Anesthesia for Non-obstetric Surgery

**DOI:** 10.7759/cureus.52382

**Published:** 2024-01-16

**Authors:** Silvio Tartaglia, Bruno A Zanfini, Salvatore Gueli Alletti, Gaetano Draisci, Antonio Lanzone

**Affiliations:** 1 Dipartimento di Scienza Della Salute Della Donna e del Bambino e di Sanità Pubblica, Fondazione Policlinico Universitario Agostino Gemelli IRCCS (Istituto di Ricovero e Cura a Carattere Scientifico), Rome, ITA; 2 Dipartimento di Scienze dell'Emergenza, Anestesiologiche e Della Rianimazione, Fondazione Policlinico Universitario Agostino Gemelli IRCCS (Istituto di Ricovero e Cura a Carattere Scientifico), Rome, ITA

**Keywords:** electronic fetal monitoring, cesarean section (cs), doppler ultrasound, general anesthesia, fetal heart rate

## Abstract

Fetal heart rate monitoring during general anesthesia for non-obstetric surgery at viable gestational ages is recommended to evaluate fetal well-being during the intervention. Alteration induced by anesthetic drugs could mimic fetal acute hypoxia, leading to pointless Cesarean sections. We report a case of a pregnant woman in the third trimester undergoing neurosurgical surgery. The continuous heart rate registration showed a non-reassuring pattern, potentially inducing the multidisciplinary team to expedite the delivery. The seriate fetoplacental Doppler evaluations were reassuring about normal fetal conditions, suggesting that ultrasound surveillance could be more reliable than intraoperative heart rate monitoring.

## Introduction

Although rare, surgery for non-obstetric reasons in pregnant women represents a non-negligible percentage (1-2%) [1]. The underlying indication for intervention and the use of general anesthesia always involve risks to the fetus. When clinical conditions are critical, maternal health must always be safeguarded, and surgery may be carried out regardless of the trimester, ensuring the most careful multidisciplinary assistance [2]. When the fetus is pre-viable, available guidelines recommend registering the fetal heartbeat before and after the procedure. At a viable gestational age, continuous monitoring of fetal heart rate (FHR) is recommended, and an urgent cesarean section should be performed in the case of non-reassuring fetal conditions if it does not interfere with the primary surgery [3]. Alterations in FHR during surgical procedures in pregnant women could be directly related to maternal hypoxia or linked to the use of anesthetics. The continuous registration during maternal general anesthesia could be misleading. Anesthetic drug-induced alterations of the fetal heart rate could simulate fetal acute hypoxia and induce unnecessary Cesarean sections (C-sections). We herein report our considerations about fetal well-being monitoring during a neurosurgical emergency intervention under general anesthesia in a pregnant woman at a viable gestational age.

This article was previously presented as a meeting abstract at The International Society of Ultrasound in Obstetrics and Gynecology (ISUOG)’s 32nd World Congress on September 16, 2022.

## Case presentation

A 37-year-old primigravida with a singleton pregnancy at 28 weeks of gestation was admitted to the emergency room due to sudden-onset headache, loss of consciousness, vomiting, aphasia, and left hemiparesis. The personal and medical history of the patient were noncontributory. Cranial computed tomography revealed a right temporal-parietal hematoma (maximum diameter of 8 cm), and an urgent neurosurgical evacuation was planned. The cardiotocography before the intervention showed a normal baseline and variability of the FHR, confirming normal oxygenation. An ultrasound scan revealed a normally developed fetus for gestational age (estimated fetal weight 1200 g, 50th percentile according to Hadlock et al. [4]) and a normal volume of amniotic fluid.

**Figure 1:**
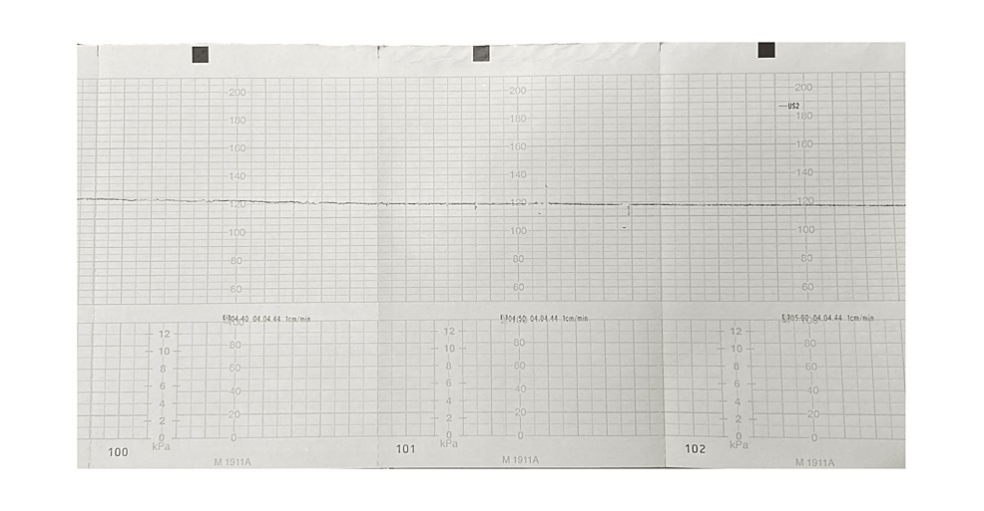
The fetal heart rate shows a silent pattern, with a complete absence of long-term and short-term variability.

A supine position with left uterine displacement was adopted in the operating room, and an additional abdominal operative field was set in case of an emergency C-section. The team was composed of two neurosurgeons, an anesthesiologist, a gynecologist, a pediatrician, two midwives, and three nurses. Anesthesia was induced using propofol 2 mg/kg, remifentanil 0.1 mcg/kg/min in an intravenous (IV) continuous infusion, and rocuronium bromide 40 mg IV. After orotracheal intubation, anesthesia was maintained with nitrous oxide 50% in oxygen, sevoflurane (1.5-2%), and fentanyl (total dose 0.3 mg IV). FHR was continuously monitored with computerized cardiotocography (cCTG). Additionally, the pulsatility index (PI) of the umbilical artery (UA), middle cerebral artery (MCA), and ductus venosus (DV) was assessed every 15 minutes using Doppler velocimetry. Maternal mean arterial blood pressure was kept steady at approximately 70 mmHg. The patient was moderately hyperventilated (arterial carbon dioxide pressure about 33 mmHg, arterial oxygen pressure at 137-160 mmHg). No relevant maternal hypotension was reported during the sedation. Continuous FHR monitoring revealed a complete loss of long- and short-term variability (STV <1.5), with a normal baseline (mean 155 bpm). No acceleration, decelerations, or uterine contractions were reported (Figure 1). Seriate Doppler evaluation started immediately after anesthesia induction and was repeated every 15 minutes, showing normal mean velocimetry for UA (PI 1.07), MCA (PI 3.1), and DV (PI 0.58) with a positive A-wave and normal cerebroplacental ratio (CPR). Reassuring Doppler results testified to the fetus's well-being, even in the presence of absent long- and short-term variability, avoiding an unnecessary C-section at a very preterm gestational age. The neurosurgical evacuation of the hematoma due to spontaneous rupture of an arteriovenous malformation was uneventful. The duration of the intervention was 150 minutes. The patient was sedated overnight with propofol 25 mg/hr and parenteral Remifentanil, and artificial ventilation was continued until the next morning. After 12 hours, a cCTG showed a normal FHR pattern. Ten days after the surgery, the patient was discharged home. After monthly ultrasound evaluations, an elective cesarean section for neurosurgical indication was performed at term, and a healthy 2970 g female newborn, with an Apgar score of 9 and 10 at the first and fifth minute, respectively, was delivered. The neonate showed normal developmental milestones at a six-month after-birth evaluation.

## Discussion

The successful management of our case leads to important considerations about intraoperative fetal monitoring. According to the American Society of Anesthesiologists (ASA) and the American College of Obstetricians and Gynecologists (ACOG), while performing non-obstetric maternal surgery [3] at a viable gestational age, intraoperative continuous electronic fetal monitoring in the presence of an obstetrician should be provided. A recent review reported a relevant number of C-sections performed during general anesthesia due to non-reassuring fetal conditions [5], with loss of variability being the most frequently reported alteration. Reduced variability is generally considered a sign of fetal hypoxia/acidosis, albeit it can also occur in cases of previous cerebral injury, infections, or drugs. Although it is well known that narcotic analgesics are related to reduced variability [6], there are still many misdiagnoses. Following guidelines for cardiotocography interpretation, prolonged absent variability should be considered a sign of impaired fetal well-being [7]. This “silent pattern” could bring about misinterpretation and unnecessary emergency cesarean sections during non-obstetric surgery. Seriate Doppler evaluations, instead, provide more reliable information about the real-time conditions of fetal oxygenation and its well-being, as the case presented here testifies.

The rationale behind the management of this case is derived from in vivo animal studies evaluating the effects of induced acute hypoxia on fetal circulation [8]. Increased DV pulsatility seems to be the first sign of hypoxemia after in-utero premature cord occlusion [9]. This is directly related to fetal oxygen tension, a significant regulator of DV diameter and, consequently, of placenta-derived blood flow. A similar effect has been described in UA [10], with a progressive decrease in diastolic velocity, increased vascular resistance, and a drop in CPR, one of the most sensitive indicators of fetal blood oxygen concentrations.

## Conclusions

Although this is a single case report with no adverse outcomes reported, it suggests that fetoplacental Doppler velocimetry plus continuous registration of the FHR by cardiotocography compared to cardiotocography alone is a more reliable tool for fetal surveillance during non-obstetric surgery under general anesthesia. The multidisciplinary team is essential to guarantee the safety of the mother-fetus dyad and to expedite immediate delivery in case of non-reassuring fetal conditions. Even if further evaluations are needed to validate this additional approach for intraoperative assessment of fetal well-being, Doppler velocimetry could help avoid unnecessary preterm cesarean delivery for suspected fetal distress.
